# The molecular mechanisms supporting the homeostasis and activation of dendritic epidermal T cell and its role in promoting wound healing

**DOI:** 10.1093/burnst/tkab009

**Published:** 2021-06-23

**Authors:** Cheng Chen, Ziyu Meng, He Ren, Na Zhao, Ruoyu Shang, Weifeng He, Jianlei Hao

**Affiliations:** 1 State Key Laboratory of Trauma, Burns, and Combined Injury, Institute of Burn Research, the First Affiliated Hospital of Army Medical University (the Third Military Medical University), Chongqing Key Laboratory for Disease Proteomics, Chongqing, 400038, China; 2 NHC Key Laboratory of Hormones and Development, Tianjin Key Laboratory of Metabolic Diseases, Tianjin Medical University Chu Hsien-I Memorial Hospital & Tianjin Institute of Endocrinology, Tianjin Medical University, Tianjin, 300134, China; 3 Department of General Surgery, Tianjin Medical University General Hospital, Tianjin Medical University, Tianjin, 300052, China; 4 Zhuhai Institute of Translational Medicine, Zhuhai People's Hospital Affiliated with Jinan University, Jinan University, Zhuhai, 519000, Guangdong, China; 5 The Biomedical Translational Research Institute, Faculty of Medical Science, Jinan University, Guangzhou, 510632, Guangdong, China

**Keywords:** Dendritic epidermal T cells, Wound healing, γδ T cells, Cytokines, Cytokine receptors, Signalling pathways, Skin

## Abstract

The epidermis is the outermost layer of skin and the first barrier against invasion. Dendritic epidermal T cells (DETCs) are a subset of γδ T cells and an important component of the epidermal immune microenvironment. DETCs are involved in skin wound healing, malignancy and autoimmune diseases. DETCs secrete insulin-like growth factor-1 and keratinocyte growth factor for skin homeostasis and re-epithelization and release inflammatory factors to adjust the inflammatory microenvironment of wound healing. Therefore, an understanding of their development, activation and correlative signalling pathways is indispensable for the regulation of DETCs to accelerate wound healing. Our review focuses on the above-mentioned molecular mechanisms to provide a general research framework to regulate and control the function of DETCs.

Highlights

- The development of DETCs and its migration and activation are summarized comprehensively.
- Molecular mechanisms which regulate the function of dendritic epidermal T-cells to control wound healing are elucidated.

## Background

T cells are divided into 2 types, αβ T cells and γδ T cells, according to the T lymphocyte receptor (TCR). γδ T cells account for approximately 1–5% of T cells and are widely distributed in the digestive tract, skin, mucosa and subcutaneous tissues. γδ T cells are a bridge between innate and adaptive immune responses and have unique biological characteristics and functions. Different types of antigens stimulate γδ T cells in a major histocompatibility complex (MHC)-independent manner to secrete a large number of cytokines and chemokines that regulate other immune and non-immune cells. According to the different TCR γ chains of γδ T cells, γδ T cells in mice include 7 subsets in total—Vγ1 to Vγ7 T cells—with different tissue specificities [1]. In contrast to murine cells, γδ T cells in humans chiefly consist of Vδ1γδ T cells and Vδ2γδ T cells. Vδ1γδ T cells are primarily distributed in the dermis, with a small number being present in the epidermis, whereas Vδ2 TCRs are expressed in peripheral blood and dermal γδ T cells [2,3]. Because Vδ1γδ T cells do not have a dendritic morphology in the human epidermis, these cells may not be called DETCs [4,5]. However, insulin-like growth factor (IGF)-1, which is secreted by Vδ1γδ T cells, also participates in wound healing, which indicates that the DETCs of mice and human Vδ1γδ T cells share the same biological function.

**Figure 1.**
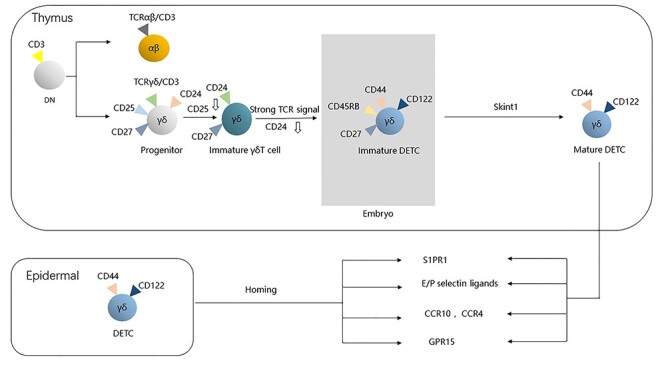
The maturation and migration of dendritic epidermal T cells (DETCs). In the thymus, γδT cells arise from a common CD4^−^ CD8^−^ double-negative (DN) progenitor. During the process of DETC maturation, CD25 expression increases and CD24 expression decreases. After T cell receptor (TCR) rearrangement, DETCs become the first wave to be produced. Positive selection adjusted by selection and upkeep of intraepithelial T cells 1 (Skint-1) determines the maturation of DETC precursors in the foetal thymus and the expression of skin-homing receptors. Homing receptors including CCR10, CCR4, G protein-coupled receptor-15 (GPR15) and E/P-selectin ligands mediate the homing of DETCs. DETCs are finally implanted in the epidermis and constantly replenished without supplement from bone marrow. *S1PR1* sphingosine l-phosphate receptor 1

**Figure 2.**
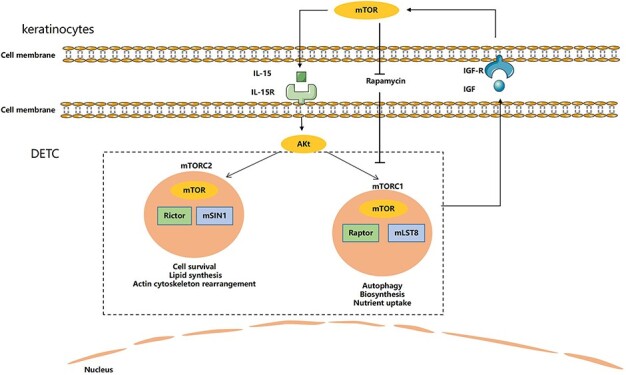
The dendritic epidermal T cell (DETC)-dependent mammalian target of rapamycin (mTOR) signalling pathways. There is a correlated loop link to mTOR, interleukin (IL)-15 and insulin-like growth factor (IGF). mTOR interacts with 2 specific target protein complexes, mTOR complex 1 (mTORC1) and mTOR complex 2 (mTORC2). Akt/mTORC1 is involved in the axis of IL-15 and IGF. A decreased level of IGF-1 leads to inhibition of mTOR activity, and a reduction in mTOR activity further induces a decrease in IL-15 production, which disturbs the homeostasis of DETCs and delays wound healing. mTOR partially participates in IL-15-mediated IGF-1 production by DETCs

According to Tonegawa’s nomenclature, DETCs specifically express Vγ5Vδ1 TCR (also called Vγ3Vδ1 TCR according to Garman’s nomenclature) in the epidermis of mouse skin, which accounts for approximately 90% of epidermal T cells [4,6,7]. The phenotype of DETCs is generally Thy-1^+^ Vγ5^+^Vδ1^+^ CD4^−^ CD8^−^ [8,9]. Thymic selection determines γδ T cell differentiation. DETCs are the first emerging (the 13th day of the embryo) and maturing subset in embryonic thymus development. The γδ T cells migrate to epithelial tissues from the thymus [10–12]. Subsequently, the mature DETCs depend on self-renewal in the implanted tissues [4]. The epidermis is under constant exposure to UV light, chemicals, allergens and traumatic injury. Effective tissue repair requires the cooperation of multiple types of cells to produce a variety of growth factors that perform effector functions that orchestrate wound healing. DETCs generally have a highly dendritic shape, with dendrites that extend basally and apically. Once activated, DETCs retract their dendrites, adopt a rounded shape and secrete a range of cytokines, chemokines and tissue-specific growth factors, which leads to increased keratinocyte proliferation, recruitment of infiltrating leukocytes and the subsequent promotion of tissue repair and immune surveillance in the skin. Therefore, regulation of the development, maturation, migration and activation of DETCs will be conducive to the treatment of wound healing.

## Review

### Development and maturation of DETCs

The branch point to determine αβ T cell and γδ T cell differentiation in the thymus is a common CD4^−^CD8^–^ double-negative progenitor. After differentiation into γδ T cells, cells are divided into different subsets. TCR is the main determining element in subset differentiation and the development and maturity of DETCs.

#### TCR rearrangement

Rearrangement of the J segment of TCR γ genes expresses Vγ 5, to induce DETC precursor cells to become the first wave of γδ T cells to arise in the foetal thymus. Finally, the DETC precursors are rearranged into invariant Vγ 5-Jγ 1Cγ 1 and Vδ 1-Dδ 2-Jδ 2Cδ. The rearrangement of TCRδ significantly affects DETC development in mice with different background strains [13].

The γδ T cells are deprived of junctional diversity. This invariant TCR allows DETCs to have innate and acquired immune cell characteristics. Some researchers have compared the number of DETCs in mice with different strain background and different locations. Different strains of mice have different numbers of DETCs [15]. The number of DETCs varies between the ear, back and footpad, even in the same mouse [16]. TCRδ rearrangement deficiency may partially explain the different numbers of DETCs across strain differences [13].

#### Positive selection

αβ T cells will experience both positive and negative selection during development. In the thymus cortex, double positive (CD4^+^CD8^+^) precursors bind with MHC I/II on thymic stromal cells to differentiate into single positive (CD4^+^CD8^−^/CD4^−^CD8^+^) T cells and precursors without useful TCR will die, which called positive selection. After positive selection, αβ T cells need to avoid becoming autoreactive T cells in negative selection. Single positive T cells will undergo apoptosis if bind with MHCI/II on macrophage or dendritic cell [17]. Unlike αβ T cells, γδ T cells in foetal mice only experience positive selection. The reason for no negative selection may be the highly fixed specificity for self-ligand, which is contrary to γδ T cell from adult. For positive selection, the data show that DETCs are selected without MHC and are CD1-restricted [18]. And selection and upkeep of intraepithelial T cells protein 1 (Skint-1) may determine the positive selection of DETC as a ligand. Skint-1 is a member of a new immunoglobulin superfamily gene cluster that encodes a 364 amino acids protein including 1 signal sequence, 1 IgV domain, 1 IgC domain, 3 transmembrane domains and 1 short cytoplasmic carbon terminal. Skint-1 is the first surface ligand related to the positive selection of γδ T cells and is primarily expressed by medullary thymic epithelial cells from the late stages of the embryonic thymus to adulthood [4,19,20].

Skint-1-deficient mice showed a loss of subcutaneous γδ T cells and the implantation of Skint-1 into the embryo increased the number of subcutaneous γδ T cells [21]. These results suggest that Skint-1 selectively regulates and restores Vγ5Vδ1^+^ DETC maturation [19]. Skint-1 directly participates in the interactions between receptors and ligands on the cell surface. The complementarity determining regions-like loop of immunoglobulins within the membrane-distal variable domain of Skint-1 is essential for Skint-1 mediation of the selection of DETC progenitors [22]. Whether Skint-1 directly interacts with TCR or other molecules on DETCs or indirectly regulates the expression of other molecules that promote the development of T cells is not clear. Other ligands may be involved in the positive selection of DETCs [23,24]. Skint-1 is the only gene specifically expressed in mice but absent in humans, which may lead to differences in TCR chain development between humans and mice [21].

### Migration of DETCs

Positive selection is not the only link with the development of DETC, but also the migration of DETC by influencing the expression of skin-homing receptors. At embryonic day 15–16, the developed and mature DETCs begin migrating from the thymus to the skin gradually through the peripheral blood circulation as the only γ δ T cells implanted in the epidermis [11]. DETC precursor cells upregulate specific homing receptors, such as sphingosine l-phosphate receptor 1 (S1PR1), chemokine receptor CCR10, CCR4, G protein-coupled receptor-15 (GPR15) and E/P selectin ligand, which are essential for the migration of DETCs.

#### Emigration from thymus

To arrive at skin, The first step of DETC is exiting the thymus. DETC precursors downregulate the thymus-retaining receptor CCR6 and upregulate S1PR1, Both of them are essential for exiting the thymus and transforms the phenotype into CCR10^high^α_E_β7^high^CCR7^low^CD62L^low^ CCR9^low^α4β7^low^ [11,25–26]. Previous studies have shown that peripheral T cells were completely absent and B cells were significantly reduced in mice whose hematopoietic cells lack a single S1PR1, for immune cells cannot emigrate from the thymus, which is essential for both αβT and γδT cells to egress from the thymic [27]. The ligand for CCR6, CCL20, is expressed in thymus and dermal tissue, which indicates that CCR6 is not only involved in thymic egress but also epidermal localizations. Without downregulation of CCR6, DETCs will improperly distribute in the dermis [28].

#### Immigration to the epidermis

After the cells leave the thymus, E/P selectin ligands and chemokine receptors determine the terminal location of DETCs. E/P selectin ligands are expressed in mature DETCs and foetal thymus precursor cells. The α 1,3 fucosyltransferase IV and VII knockout mice cannot synthesize the carbohydrate motifs of E/P selectin ligands. DETCs in these mice are unable to migrate effectively to the epidermis, which indicates that E/P selectin ligands are important for the peripheral migration of DETCs [29–30]. Jiang [30] reported that the development of foetal thymus DETCs was normal in CCR4 knockout mice but the number of DETCs in mouse epidermis was significantly reduced, which suggests that CCL17 (a CCR4 ligand) provides direction for DETCs. However, the number of DETCs in CCR10 knockout mice was normal. Contrary to Jiang, Yan [26] believes that CCR10 is crucial for the migration and location of DETCs, especially from dermis to epidermis. DETCs are increased in dermal regions in CCR10 knockout mice and decreased in epidermal regions. Unlike CCR10, another skin-homing receptor, GPR15, is responsible for recruiting DETCs from thymus to skin, for which GPR15 knockout mice showed diminishment both in dermal and epidermal. Meanwhile, the influence of GPR15 can be compensated for by CCR4 and CCR10 [31,32].

#### Implantation in the epidermis

After arrival in the epidermis of the skin, DETC dendrites are anchored on the top epidermis of keratinocytes via special ligands that are recognized by the γ δ TCR receptor and E-cadherin receptor integrin α E β 7 [33]. The remaining DETC dendrites project to the basal epidermis, and their extension and contraction are highly mobile. The epidermal keratinocytes around the dendritic processes primarily express E-cadherin [34,35] and DETCs may express CD103, which is enriched at the end of the dendritic processes and acts as an anchor for the process via binding with E-cadherin. DETCs that have migrated to the epidermis are stably implanted in it via the interaction of cell surface adhesion molecules and surrounding cells.

The existence of adhesion molecules, epidermal homing receptors and other molecules is undoubtedly crucial for the development of DETCs. How these factors precisely regulate the development of DETCs and promote their migration to the epidermis as well as the survival and epidermis retention of DETCs needs further examination, which is an important direction for future studies of DETCs.

### Homeostasis maintenance and activation of DETCs

Langerhans cells (LCs) and DETCs are the only 2 immune cell lineages in the epidermis and they share the same origin and mechanisms of homeostatic maintenance. Both cells originate from yolk sac progenitors and are clonally replenished after implantation in the epidermis. DETCs must self-renew and be activated to play biological roles. Therefore, the essential signalling molecules in the homeostatic maintenance and activation of DETCs should be emphasized.

#### TCR

The function of TCR plays critical roles in the development, survival and activation of DETCs. As far as we know, DETC activation requires 2 signals: a pre-activation signal, which is generated by TCR, can keep DETCs in an incomplete activation status, and a costimulatory signal, such as junctional adhesion molecule (JAML) or Natural killer group 2 member D (NKG2D), can completely activate DETC. TCR signalling maintains the expression of CD69 and CD122, which results in DETC pre-activation, because CD69 and CD122 are activation markers of DETCs.

DETCs establish a polarized conduit system for transepithelial cargo transport via TCR activation in the steady state, which may help to accumulate mature lysosomes and probe the molecular composition of the epidermis for communication with the dermis [36]. However, in the stressed state, trauma and inflammation may induce TCR redistribution [37]. Linker for activation of T cells (LAT) is an essential signaling molecule downstream of TCR. Depletion of LAT gene depletion in the process of wound healing leads to impairment of TCR signaling and DETC activation, which further results in delayed wound healing [38]. TCR may also serve as a bridge between DETCs and keratinocytes. Keratinocyte-responsive γδ TCR is indispensable for DETC activation and homeostasis maintenance in the epidermis during wound healing [39]. Further investigation of natural ligands of TCR should be emphasized to fully elucidate the regulatory mechanisms underlying the lifelong homeostatic maintenance of DETCs.

#### Costimulatory signal

Most cytokines secreted by DETCs are detected in low amounts under resting conditions, but secretion is upregulated rapidly upon stimulation. This change is, in part, due to the costimulatory signal. NKG2D, one such costimulatory signal, belongs to NKG2 family and is an important activation receptor in the innate immune system that transmits activation signals and activates the immune system by identifying ligands on target cells. NKG2D ligands include Rae-1α-ɛ, H60a-c and Mult1 in mice and MHC class I chain-related molecules A/B and UL16 binding proteins in humans [40,41]. DETCs constitutively express NKG2D, including NKG2D-S and NKG2D-L isoforms, and DAP10 and DAP12 adaptor proteins [42,43]. NKG2D is a coactivating stress receptor that directly triggers the cytotoxicity of DETCs via the phosphatidylinositol 3-kinase (PI3K)-dependent signalling pathway without cytokine production or Syk/ZAP70 activation [42]. The NKG2D ligand H60c is highly expressed on keratinocytes during the process of wound repair and activates DETCs by interacting with NKG2D that is expressed on them [45]. Blockade of the interaction between H60c and NKG2D reduces keratinocyte growth factor (KGF) secretion and delays wound healing. H60c has a more general effect on the maintenance of epithelial integrity [46]. NKG2D and NKG2D ligands participate in allergen-induced DETC activation during the process of contact hypersensitivity. Blockade of NKG2D signalling partially inhibits DETC activation, as measured by interferon-γ (IFN-γ) production and morphological changes [47].

JAML is a member of the family of connected adhesion molecules that binds to Coxsackie and adenovirus receptors to stimulate DETCs [48]. After skin injury, JAML recruits leukocytes to the wound bed and facilitates leukocyte–endothelium interactions, which contributes to the proliferation and cytokine production of DETCs [49]. Similar to the ICOS, the third member of the CD28/CTLA-4 family, mediated costimulation of αβ T cells, JAML may work during later stages of DETC activation [48].

Axon-guided factor 4D (also known as CD100) is a characteristic signalling protein on T cells. CD100 plays a vital role in humoral and cellular immunity [50]. DETCs with high CD100 expression interact with plexin B2, which actives DETCs by trigging the extracellular signal-regulated kinase (ERK) and cofilin signalling pathways. DETCs cannot be converted from a dendritic to round shape in the absence of CD100-mediated signals [51]. Impaired re-epithelialization will delay the repair of skin wound. The ligands of CD100 include plexin B1, CD72 and plexin B2. Unlike plexin B2, plexin B1 and CD72 do not affect the activation of CD100-mediated DETCs [51].

These results support the regulatory roles of NKG2D, JAML and CD100 signalling in DETC activation, cytokine secretion and antigen presentation, which contribute to skin wound healing; the regulation of DETC function and homeostasis via these signalling pathways may provide potential aids in wounds and skin diseases. In contrast, proteins such as Ly49E, CD94/NKG2A and PD-1 on DETCs may play a role in inhibiting DETC activation. Suppression of the expression of these proteins also benefits DETC activation [52]. Notably, E-cadherin and CD103 are expressed on DETCs and exert opposite functions. E-cadherin is downregulated on activated DETCs and inhibits cell activation, but CD103 mediates the short-term adhesion between DETCs and keratinocytes, which influences the motility of DETCs and provides a costimulatory signal for cytokine production in DETCs. [35].

#### Cytokines and cytokine receptors

In addition to the above-mentioned molecules, some cytokines, including interleukin (IL)-2, IL-15 and IL-7 affect the differentiation and proliferation of DETCs. IL-2 is a key factor in promoting the activation and proliferation of T cells [53]. The effects of IL-2 are mediated via IL-2 receptor, which consists of 3 subunits, the α, β and γc chains [54]. During the development of DETCs, IL-2Rβ transduces survival and expansion signals in the foetal thymus and skin. IL-2Rβ-deficient mice showed a decrease in the number of mature DETCs [55].

IL-15 is primarily produced by macrophages and epithelial cells, which can promote T cell, natural killer (NK) cell and B cell proliferation and differentiation and induce IFN-γ production. The IL-15 receptor is a trimer that shares 2 chains (β and γc) with the IL-2 receptor and has a unique α chain. Therefore, the biological functions of IL-15 are partially similar to IL-2. IL-15/IL-2Rβ signalling is also indispensable for localization and proliferation of DETCs via regulation of the expression levels of IL-2Rβ and IL-2Rγc. However, IL-15Rα (CD215), but not IL-2Rα (CD25), is highly expressed on the surface of skin located DETCs [56,57], which suggests that IL-15 is more important than IL-2 for homeostasis and the efficient activation of DETCs at the early stage of wound healing. IL-15 accelerates wound healing by promoting IGF production by DETCs [58]. The activation of IL-15 partially depends on the mammalian target of rapamycin (mTOR) pathway. Impaired mTOR pathway activation and decreased IL-15 production influence DETC homeostasis, which leads to the delay of wound healing [59,60]. The over-expression of IL-15 in the epidermis enhances innate and adaptive cutaneous immune responses in the development of contact hypersensitivity [61]. The transcription factor IFN regulatory factor-1 promotes the maturation of DETCs by regulating the expression levels of IL-15 in the skin [55,62]. These studies revealed that IL-2 and/or IL-15 play critical roles in the activation and proliferation of DETCs. Supplementation of exogenous IL-2 or IL-15 may be a new strategy to promote skin wound healing.

IL-7 is a pleiotropic cytokine that is necessary for the development, differentiation and sensitivity to apoptosis of DETCs. Keratinocytes, fibroblast mesenchymal cells, thymus and intestinal epithelial cells can secrete IL-7 [63]. The IL-7 receptor is a heterodimer that includes an α-chain and a γc-chain. IL-7 plays biological roles via binding to the IL-7 receptor, which generally activates the JAK– signal transducer and activator of transcription (STAT) and PI3K/Akt Protein Kinase B pathways [64]. IL-7 has been proven to be a key factor in the rearrangement of the TCR γ gene. IL-7 and the IL-7 receptor rearrange the TCR γ gene site via the opening of a regulatory site for V-J recombinase, which may plays a role via the JAK/STAT pathway [65]. Keratinocyte-derived IL-7 can upregulate TCR/CD3 expression of DETCs, which affects their phenotypic maturation [66]. IL-7 can also promote the survival and proliferation of DETCs, which may be enhanced in combination with TNF-α [67,68]. The IL-7 receptor may induce the initiation of V-J recombination of TCR γ genes, which is different from IL-2R and IL-15 receptor [65]. The expression levels of IL-7 are decreased in the early stage of dermal injury and increased in the middle and late stages [69]. Keratinocyte-derived IL-7 promotes the proliferation of activated DETCs during the process of wound healing, which further promotes the proliferation of resident stem cells in the tissues and accelerates wound healing [70]. IL-7 promotes the development of DETCs during the process of contact hypersensitivity by regulating the rearrangement and transcription of the Vγ5 region. IL-7 also participates in the accumulation of DETCs in the lymph nodes and at the antigen-challenge site after skin sensitization [71]. In summary, IL-7 plays substantial roles in DETC homeostasis in addition to IL-2 and IL-15. Remarkably, these cytokines serve differential functions in DETC development in the epidermis, though their receptors share some subunits and a similar set of signalling pathways.

### DETC-dependent signalling pathways during would healing

As the first barrier of skin, DETCs promote wound healing, re-epithelialization and immune cell recruitment to the wound sites. To accomplish these effects, the epidermal-resident DETCs begin to migrate to wound sites and retract dendritic tentacles within several hours. Skin γδ T cells are primed for a rapid response primarily because these cells exist in a pre-activated state. However, little is known about the signalling pathways that regulate this response. It is well accepted that mTOR, aryl hydrocarbon receptor (AhR) and STATs are partially involved in the function of DETCs.

#### The signal transducer and activator of transcription

The STAT signalling pathway plays an important role in the development and proliferation of immune cells. The STAT family is downstream of the JAK family, which is a group of proteins that function as signal transducers and transcription factors. Activated STAT enters the nucleus and regulates gene transcription, which induces extracellular signals that are transmitted to the nucleus and regulate the expression levels of target genes. STAT family members partially participate in DETC-related skin injury and wound healing [72,73]. STAT5 affects the epigenetic properties of DETCs by activating the Vγ5 promoter via binding to the HsA element and inducing its histone acetylation. STAT5 also induces Jγ1 promoter mutant mice to exhibit fewer DETCs by controlling V (D) J recombination of the TCR γ locus [74–75]. STAT3 signalling enhances the expression levels of Skint-1, which plays vital roles in maintaining the homeostasis of DETCs and facilitating wound repair in the epidermis. STAT3 phosphorylation is upregulated in the procession of IL-1β/IL-23-triggered IGF-1 production in DETCs [76,77]. STAT1 and STAT3 influence the biological functions of keratinocytes and indirectly regulate the crosstalk between keratinocytes and DETCs. Keratinocyte-specific deficiency of STAT3 impairs the wound repair and hair cycling processes. Keratinocytes may aggravate the development of vitiligo by recruiting DETCs, which is regulated by the expression of STAT1 in keratinocytes [78,79]. In summary, STAT signalling pathways are involved in the processes of skin injury and wound repair via modulation of DETC homeostasis and functions.

#### Mammalian target of rapamycin

The mTOR is a serine/threonine kinase protease that forms 2 structurally and functionally distinct interactions with 2 specific target protein complexes, mammalian target of rapamycin complex 1 (mTORC1) and mTORC2. mTORC1 consists of 3 cores, mTOR, Raptor and mLST8, which regulate protein and ribosome biosynthesis, nutrient uptake and autophagy. mTORC2 is primarily composed of Rictor and mSIN1, which regulate actin cytoskeleton rearrangement, cell survival and lipid synthesis. The rapamycin–FKBP12 complex specifically inhibits mTORC1 but does not directly bind or inhibit mTORC2 [80–82].

mTOR is an important metabolic pathway that regulates some pathological and physiological processes [83]. The PI3K/Akt/mTOR signalling pathway is upregulated in psoriasis. The mTOR inhibitor rapamycin can ameliorate skin injury via inhibition of the mTOR signalling pathway [84–86]. Deficiency of Raptor-mediated mTORC1 activity impairs αβ T cell development but enhances γδ T cell development, including IL-17^+^ γδ T cells [87,88]. However, the homeostasis of DETCs is disrupted when mTOR activity is inhibited. Rapamycin-treated mice show impaired biological functions of γδ T cells, but not keratinocytes, during the process of wound repair. Impaired γδ T cells cannot proliferate, migrate or secrete normal levels of growth factors, which leads to delayed wound healing [89]. Keratinocyte-derived IL-15 is necessary for the development and homeostasis of DETCs in the epidermis, and DETCs further promote wound repair by producing IGF-1 and KGF. Notably, IL-15, IGF-1 and mTOR are interconnected and block DETC homeostasis in diabetes. A decreased level of IGF-1 leads to inhibition of mTOR activity and the reduction of mTOR activity further induces a decrease in IL-15 production, which disturbs the homeostasis of DETCs and delays wound healing. mTOR partially participates in IL-15-mediated IGF-1 production by DETCs [58,59]. However, recent studies have indicated that a low dose of rapamycin increases the expression levels of IL-15 and IGF-1 to promote wound healing via activation of the Akt/mTORC2 pathway. In contrast, a high dose of rapamycin inhibits the expression levels of IL-15 and IGF-1, which hinders the wound healing process [60].

These results show that the homeostasis of DETCs is disrupted after the inhibition of mTOR activity. The mTOR signalling pathway has multiple feedback loops and complementary feedback loops promote cell survival and proliferation. A deep understanding of the molecular mechanism of the mTOR pathway and its feedback loops may provide new methods for the treatment of skin injury via the regulation of DETC homeostasis.

#### Aryl hydrocarbon receptor

The AhR is a cytoplasmic transcription factor that is primarily activated by ligands. Synthetic and natural ligands for AhR were discovered. Synthetic ligands, such as aromatic hydrocarbons, possess a greater affinity for the receptor but are more toxic. Natural ligands with less toxicity and are derived from the essential amino acid tryptophan [90]. AhRs are widely distributed in various tissues, such as lung, liver, kidney, tonsil and skin. In the epidermis, AhR also plays an essential role in the postnatal maintenance of skin-resident DETCs by directly modulating c-Kit expression, which provides a potential link between AhR and wound healing [91]. AhR activation inhibits wound repair and AhR downregulation promotes wound repair [92–93]. AhR knockout studies indicate that the deficiency of AhR impairs the proliferation and alters the morphology of DETCs, which leads to a decreased secretion of granulocyte-macrophage colony-stimulating factor (GM-CSF). Because DETCs are the main source of GM-CSF in the epidermis, and GM-CSF is required for the maturation of LCs, AhR deficiency would impair their maturation and suppress the corresponding immune responses [91]. Microarray RNA profiles of DETC demonstrated that the inflammatory activity of DETCs was mostly related to AhR-dependent regulatory pathways, which further demonstrates the important role of AhR in maintaining the homeostasis of DETCs [94]. In summary, many studies have revealed that AhR regulates the proliferation of DETCs and plays critical roles in skin injury.

Skin is a rich source of DETCs, which suggests that the maintenance of DETC homeostasis is critically involved in cutaneous defence and wound repair. TCR, Skint-1, AhR, IL-2, IL-15, IL-7 and their receptors are also required for the survival and full activation of DETCs. Notably, recent research has shown that Skint family members, including Skint3 and Skint9, also affect re-epithelialization in wound healing. These proteins mediate keratinocyte–DETC crosstalk via IL-6/STAT3 signalling to regulate the behaviour of DETCs [76]. A deep understanding of the roles of these molecules in epidermal DETC homeostasis will shed light on skin wound healing. However, additional studies are needed to identify other factors that may be relevant to the homeostasis of DETC.

### The role of DETC in wound healing

As discussed above, DETCs transform into round cells after skin injury and the level of secreted cytokines is increased. Current research on DETCs is primarily focused on the inflammatory and growth phases, but not the remodelling phase of wound healing.

#### Re-epithelialization

Re-epithelialization is a crucial factor during the growth phase and it determines the terminal speed of the entire wound healing process. The epidermis consists of a basal layer, a spinous layer, a granular layer and stratum corneum, from the inside out. The basal layer is composed of stem cells that proliferate and differentiate into keratinocytes that migrate to the wound surface to compensate for cell loss. Consequently, the regulation of these activities of keratinocytes also regulates the speed of wound healing [95].

The epidermis is constantly renewing in the resting state to maintain skin homeostasis. Sharp [96] reported that IGF-1 secreted by DETCs plays an essential role in the homeostasis of apoptosis and differentiation of keratinocytes. The re-epithelialization of injured skin is also inseparable from IGF-1. IGF-1 is a growth hormone with peptides that are structurally and functionally homologous to insulin and it regulates the survival, migration, self-renewal and differentiation of various cells. IGF-1 is a hormone and a cytokine that plays biological roles via binding to the IGF-1 receptor [97–98]. Notably, studies have demonstrated that DETCs express IGF-1 and the IGF-1 receptor, which are involved in homeostatic epidermal maintenance. IGF-1 secretion from DETCs reduces apoptosis by inducing the expression of c98, a novel gene homologous to bcl-2, which promotes the survival of keratinocytes by secreting cytokines to accelerate wound repair [99,100]. Decreased IGF-1 expression was detected at wound sites in both diabetic animals and in patients with delays in wound healing [100–104]. IGF-1 also inhibits the differentiation of keratinocytes to maintain the stemness of epidermal cells via the PI3K, ERK1/2 and mTOR signalling pathways [105]. In contrast to IGF-1, previous studies reported that increased levels of IGF-2 in the basal layer of the epidermis led to delayed wound repair [104]. Relative to the homologous sequence of IGF-1, there are few studies on the role of IGF-2 in wound healing. Whether DETCs secrete IGF-2 and the balance between IGF-1 and IGF-2 in the epidermis are topics worthy of further investigation. There is also an IL-1β/IL-23–IL-17A axis between DETCs, Vγ4γδ T cells and keratinocytes. IL-17A, produced by Vγ4γδ T cells, enhances the expression of IL-1β and IL-23 in the epidermis, which indirectly inhibits IGF-1 production by DETCs and leads to delayed wound repair.

In addition to IGF-1, DETCs regulate the biological functions of keratinocytes and rebuild the skin protective barrier by secreting KGF, which is a member of the fibroblast growth factor (FGF) family. Activated DETCs produce KGF-1 (FGF-7) and KGF-2 (FGF-10), which bind to the KGF receptor. Then, phosphorylated KGF receptor activates downstream signalling pathways, including PI3K/Akt, mTOR and ERK–mitogen-activated protein kinases (MAPK), to promote epidermal cell proliferation [106–112]. However, unlike IGF, which was constitutively produced by DETCs at low levels [100], KGF is secreted only when stimulated because the resting epidermis does not constitutively express TCR ligands in DETCs. When the epidermis is damaged, keratinocytes around the wound sites rapidly upregulate their expression of DETC TCR ligands, which activates adjacent DETCs immediately to secrete KGF in large quantities. KGF further acts on keratinocytes to accelerate the process of wound healing by stimulating their migration and proliferation [46,101,113,114]. Mechanistic research indicates that DETC-derived KGF stimulates keratinocytes to produce hyaluronan, which controls macrophage recruitment to the wound sites. This mechanism partially explains the accumulation of inflammatory cells around the wound sites [115]. These results suggest that DETC-derived IGF-1 and KGF, serving as growth factors, play vital roles in epidermal homeostasis and wound enclosure. IGF-1 and KGF may have some synergistic effects on the promotion of wound healing and depend on the same signalling pathways.

#### Inflammation

DETCs are immune cells that also regulate the inflammatory phase of wound healing. Activated DETCs express CCL-3 (MIP-1), CCL-4 (MIP-1), CCL5 (Rantes) and XCL1 (lymphocyte chemokines), which induce the migration of inflammatory cells. XCL1 belongs to the class of XC chemokines and is the most potent and abundant chemokine produced by DETCs. XCL1 binds to XCR1 on CD8^+^ T cells to induce migration to the wound surface but it does not influence the migration of neutrophils or macrophages [116]. As mentioned above, DETCs affect the maturation of LCs via GM-CSF. DETC-derived IFN-γ inhibits the proliferation of LCs and alters their antigen-presenting capacity [91,117–119]. LCs and macrophages belong to the mononuclear phagocyte system and play critical roles in wound healing. The effect of DETCs on the recruitment of macrophages remains controversial. KGF secreted by DETCs induces keratinocytes to release hyaluronan to mediate the migration of macrophages [114]. In contrast, Rani [120] showed that γδ T cells inhibited the infiltration of myeloid cells, including macrophages. Nitric oxide (NO), a type of free radical, also acts as a second-messenger molecule. Inducible NO synthase (iNOS) in macrophages primarily produces NO during wound healing. NO facilitates re-epithelialization, wound closure and collagen deposition and recruits inflammatory cells, such as neutrophils and macrophages. γδ T cells are essential in increasing iNOS during burn injuries [121,122]. Notably, iNOS is a marker of M1 macrophages. DETCs may increase the expression of iNOS by influencing the polarization of macrophages.

## Conclusions

The development of DETCs is closely related to their function. Deficiency or impaired function of DETCs induces epidermal homeostasis imbalance and wound repair dysfunction, which leads to chronic refractory wounds, ulcers and infections. Anything that goes wrong during maturation, migration and replenishment of DETCs would lead to deficiency or impaired function of DETCs. The molecular mechanisms of DETC development will provide targets for the treatment of patients with chronic wounds. DETCs are also involved in inflammatory skin diseases, skin inflammation and skin tumour immunity. It is crucial to clarify the potential mechanisms of DETCs in these diseases.

## Abbreviations

AhR: aryl hydrocarbon receptor; CD100: axon-guided factor 4D; DETCs: dendritic epidermal T cells; ERK: extracellular signal-regulated kinase; FGF: fibroblast growth factor; GPR15: G protein-coupled receptor-15; IGF: insulin-like growth factor; IL: interleukin; iNOS: inducible nitric oxide synthase; KGF: keratinocyte growth factor; LCs: Langerhans cells; MHC: major histocompatibility complex; mTOR: mammalian target of rapamycin; NO: nitric oxide; PI3K: phosphatidylinositol 3-kinase; S1PR1: sphingosine l-phosphate receptor 1; Skint-1: selection and upkeep of intraepithelial T cells 1; STAT: signal transducer and activator of transcription; TCR: T lymphocyte receptor

## Funding

This work was supported by grants from the General Program of National Natural Science Foundation of China (31872742 to WH, 31970830 and 81630025 to JH, 31800722 to ZM); the Military Medical Science and Technology Youth Cultivation Plan (20QNPY024); the Traditional Chinese Medicine Bureau of Guangdong Province (2018071 to JH); the Guangzhou Municipal Science and Technology Bureau (201904010090 to JH); the Health Commission of Guangdong Province (A2019520 to JH); and a grant from the Tianjin Natural Science Foundation (19JCQNJC11400 to ZM).

## Authors’ contributions

CC and ZM wrote the manuscript. HR and NA helped to design the manuscript structure and write the manuscript. WH and JH evaluated and reviewed manuscript structure, ideas and science. All authors read and approved the final manuscript.

## Conflicts of interest

The authors have no conflicts of interest to declare.
